# Draft genome sequences of the type strains of *Shigella flexneri* held at Public Health England: comparison of classical phenotypic and novel molecular assays with whole genome sequence

**DOI:** 10.1186/1757-4749-6-7

**Published:** 2014-03-31

**Authors:** Philip M Ashton, Kate S Baker, Amy Gentle, David J Wooldridge, Nicholas R Thomson, Timothy J Dallman, Claire Jenkins

**Affiliations:** 1 Gastrointestinal Bacteria Reference Unit, Public Health England, 61 Colindale Ave, NW9 5HT London, England; 2 Wellcome Trust Sanger Institute, Genome Campus, Hinxton CB10 1SA, UK; 3 Department for Bio-analysis and Horizon Technologies, Next Generation Sequencing, Public Health England, 61 Colindale Ave, London NW9 5HT, England

**Keywords:** *Shigella flexneri* type strains, Next generation sequencing technology, Molecular serotyping

## Abstract

**Background:**

Public Health England (PHE) holds a collection of *Shigella flexneri* Type strains isolated between 1949 and 1972 representing 15 established serotypes and one provisional type, E1037. In this study, the genomes of all 16 PHE Type strains were sequenced using the Illumina HiSeq platform. The relationship between core genome phylogeny and serotype was examined.

**Results:**

The most common target gene for the detection of *Shigella* species in clinical PCR assays, *ipaH*, was detected in all genomes. The type-specific target genes were correctly identified in each genome sequence. In contrast to the *S. flexneri* in serotype 5 strain described by Sun *et al*. (2012), the two PHE serotype 5 Type strains possessed an additional *oac* gene and were differentiated by the presence (serotype 5b) or absence (serotype 5a) of *gtrX*. The somatic antigen structure and phylogenetic relationship were broadly congruent for strains expressing serotype specific antigens III, IV and V, but not for those expressing I and II. The whole genome phylogenies of the 15 isolates sequenced showed that the serotype 6 Type Strain was phylogenetically distinct from the other *S. flexneri* serotypes sequenced. The provisional serotype E1037 fell within the serotype 4 clade, being most closely related to the Serotype 4a Type Strain.

**Conclusions:**

The *S. flexneri* genome sequences were used to evaluate phylogenetic relationships between Type strains and validate genotypic and phenotypic assays. The analysis confirmed that the PHE *S. flexneri* Type strains are phenotypically and genotypically distinct. Novel variants will continue to be added to this archive.

## Background

*Shigella flexneri* is the predominant cause of shigellosis in the developing world [1], making appropriate subtyping tools for tracking *S. flexneri* epidemiology vital to global public health. The *S. flexneri* serotyping scheme differentiates isolates serologically based on the expression of the major type specific somatic antigen (I-VI) and common group factor antigens (3,4 designated Y and 7,8 designated X) [2]. The common group factor antigens account for the complex intra-serotype relationships. Currently, there are 15 established serotypes. Traditional *S. flexneri* serotyping is performed by slide agglutination using antiserum raised in rabbits against type specific and group factor antigens. Recently, Sun *et al.*[3] published a multiplex PCR approach for molecular serotyping of *S. flexneri*. This method differentiates the 15 accepted serotypes based on known differences in (i) their *gtr* genes encoding the type specific antigens I, II, IV, and V, group factor antigen 7,8 (X) and 1c (*gtrI*, *gtrII*, *gtrIV*, *gtrV*, *gtrX*, and *gtrIC*) (ii) the *oac* gene that mediates O-acetylation modification in serotypes 1b, 3a, 3b, and 4b and (iii) the *wzx*_*6*_ for detection of serotype 6.

Public Health England (PHE) holds an historic collection of 16 *S. flexneri* Type strains isolated between 1949 and 1972. Strains belonging to this set have been used to produce standardised antiserum for the phenotypic serotyping scheme at PHE for over 60 years. To increase the utility of this collection, we report the draft whole genome sequences of the 16 PHE *S. flexneri* Type strains in order to facilitate a greater understanding of how whole genome phylogenies compare to typing data generated from diagnostic and molecular serotyping targets.

## Methods

### Bacterial strains

The 16 strains of *S. flexneri* analysed in this study are shown in Table 1. Strains used in this study were serotyped by slide agglutination using both commercially available monovalent antisera (Denka Seiken, Japan) and monoclonal antibody reagents (Reagensia AB, Sweden) and in-house antisera raised in rabbits [4] to all type specific somatic antigens and the group factor antigens. All strains were tested using the PCR serotyping assay described by Sun *et al*. [3].

**Table 1:** Comparison of the phenotypic and genotypic serotyping

**Phenotypic results**		**PCR results**		**Genome sequencing results**		
**Agglutination reactions**	**Phenotypic serotype [**[2]**]**	**Genes detected using PCR scheme [**[3]**].**	**PCR serotype [**[3]**]**	**Serotype derived from genome sequence according to PCR scheme [**[3]**]**	**Number of SNPs different in the core regions compared with reference strain**	**Proportion of reference mapped to**
Type I	1a	*gtrI*	1a	1a	2549	0.88
Type I + Group 6	1b	*gtrI* + *oac*	1b	1b	2552	0.87
MASF 1c	1c	*gtrI* + *gtrC*	1c	1c	2661	0.88
Type II + Y	2a	*gtrII*	2a	2a	731	0.91
Type II + X	2b	*gtrII* + *gtrX*	2b	2b	2650	0.89
Type III + X	3a	*gtrIII* + *gtrX*	3a	3a	6947	0.86
Type III + Y	3b	*gtrIII*	3b	3b	6976	0.85
Type III + Y	3c	*gtrIII*	3c Not included in the PCR scheme	Not included in the PCR scheme	7030	0.85
Type IV + Y	4a	*gtrIV*	4a	4a	2595	0.87
Type IV + Group 6	4b	*gtrIV* + *oac*	4b	4b	2528	0.87
Type V + Y	5a	*gtrV* + *oac*	5a Not included in the PCR scheme	Not included in the PCR scheme	4388	0.89
Type V + X	5b	*gtrV* + *oac* + *gtrX*	5b Not included in the PCR scheme	Not included in the PCR scheme	4294	0.88
Type VI	6	*wzx6*	6	6	47787	0.74
MASF IV-1 E1037	E1037	*gtrIV* + *lpt-O*	MASF IV-1 E1037	4a with Ipt-O (opt) gene	3333	0.88
X	X	*gtrX*	X	X	6937	0.84
Y	Y	No amplification	Y	Y	752	0.92

### Genome sequencing and analysis

Genomic DNA was isolated from an overnight culture using the Wizard kit (Promega, Madison, Wisconsin, USA) and was sequenced at the Wellcome Trust Sanger Institute (WTSI) and PHE. Paired end libraries where each pair was 100 bp in length were generated on the Illumina Hiseq 2500 instrument (San Diego, California, USA). Resulting FASTQ reads were processed using Trimmomatic v0.27 [5] to remove bases with a PHRED score of less than 30 and read length less than 50 bp after quality trimming. High quality reads were then mapped to the reference strain, *S. flexneri* serotype 2a strain 2457 T (AE014073.1) [6], using BWA v0.6.2 and Single Nucleotide Polymorphisms called using GATK v2.5.2 in Unified Genotyper mode [7]. Positions in the reference genome where GATK mapping quality was below 30 and genotyping quality was below 50 in any strain were excluded from further analysis. Single Nucleotide Polymorphisms (SNPs) were defined as the sub-set of high quality positions (MQ > 30, GQ > 50) where the base identified varied from the reference position. *De novo* assembly was performed using Velvet v1.2.3 [8] with K-mer selected using VelvetK (Table 2) (http://www.vicbioinformatics.com/software.velvetk.shtml). A maximum likelihood phylogenetic tree was drawn using MEGA v5.1 with 500 bootstraps based on an alignment of 10632 SNPs called against the *S. flexneri* serotype 2a strain 2457 T reference genome.

**Table 2:** **Genome statistics for the *S. flexneri *** **genomes sequenced in this study**

**Serotype**	**Number of high quality mapped reads**	**Average coverage of S. flexneri 2457 T reference**	**Kmer used in Velvet assembly**	**N50**	**Number of contigs**	**De novo assembly genome size**
1a	5593126	121.5896957	79	31660	526	4369471
1b	5332110	115.9154348	79	31344	387	4176066
1c	5593956	121.6077391	81	30176	425	4255254
2a	5541229	120.4615	79	33398	417	4262526
2b	4869600	105.8608696	77	32299	421	4328906
3a	4590499	99.79345652	77	33146	510	4601423
3b	4803610	104.4263043	79	33159	450	4494495
3c	5604902	121.8456957	81	33478	451	4495552
4a	5328467	115.8362391	81	33309	413	4267213
4b	5106835	111.0181522	79	34439	391	4188134
5a	5461876	118.7364348	79	29622	443	4362146
5b	5250432	114.1398261	79	29416	459	4354058
6	6350211	138.0480652	85	20677	614	4401927
E1037	15797791	343.4302391	93	36513	378	4350496
X	17982247	390.918413	93	33586	414	4324701
Y	20943063	455.2839783	95	29717	447	4491024

### Genomic data deposition

Wellcome Trust Sanger Institute sequence data is available in the Short Read Archive under the following accession numbers (serotype): ERS088060 (1a); ERS088061 (1b); ERS088062 (1c); ERS088063 (2a); ERS088064 (2b); ERS088065 (3a); ERS088066 (3b); ERS088067 (3c); ERS088068 (4a); ERS088069 (4b); ERS088071 (5a); ERS088072 (5b); ERS088073 (6); ERS088074 (X); ERS088075 (Y); ERS088076 (E1037).

### Findings

Mapping of the sequencing reads to the 4.6 Mbp *S. flexneri* serotype 2a strain 2457 T reference genome resulted in 99–455 times coverage, with between 731 and 47787 SNPs compared to the reference genome (Table 1). *De novo* assembly resulted in an average N50 of 31621 with an average of 447 contigs (Table 2).

**Figure 1:**
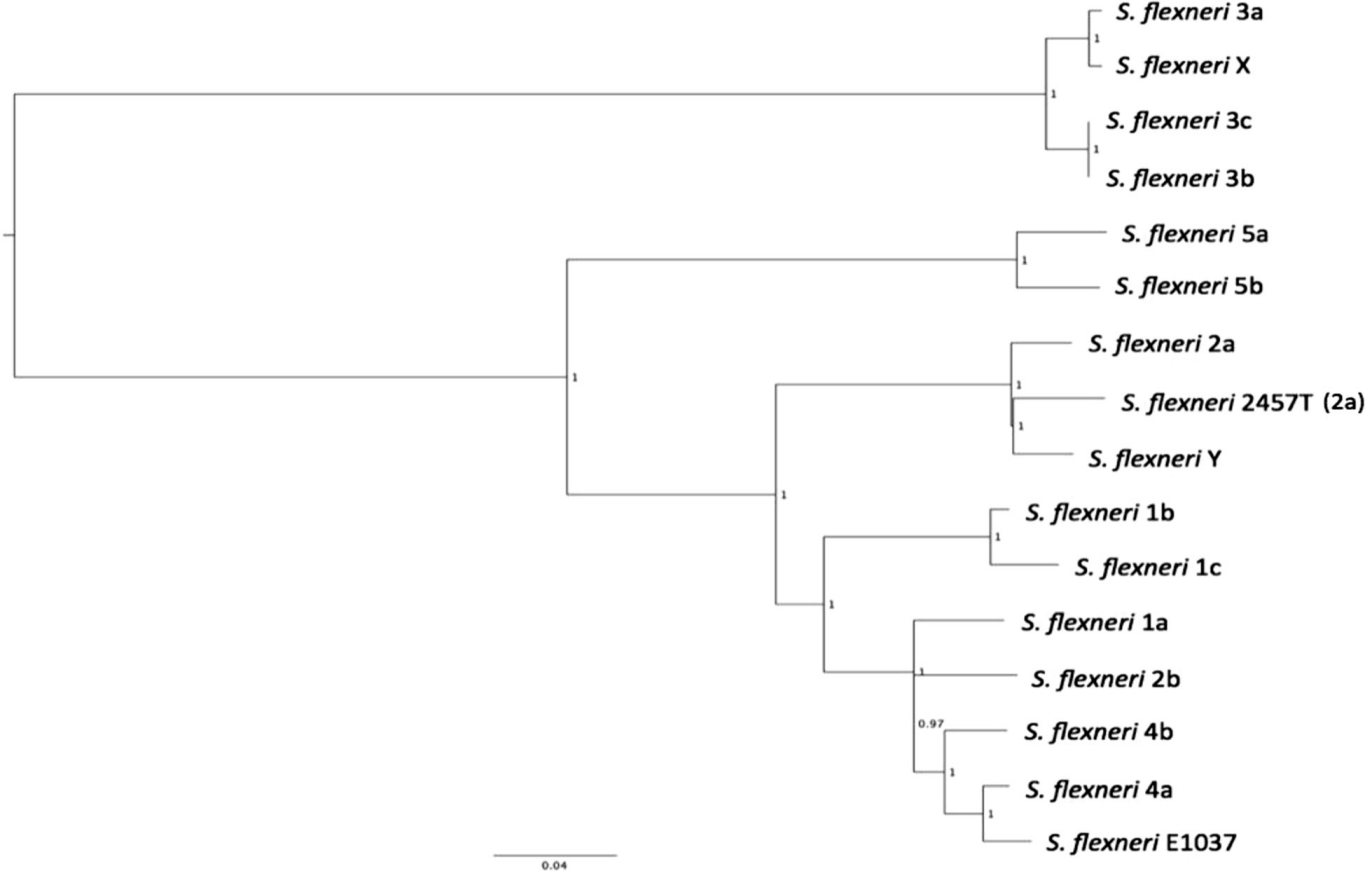
**Midpoint-rooted phylogenetic tree of *S. flexneri *type strains based on 10631 variant positions in the core genome, node labels are bootstrap values based on 500 bootstraps.***S. flexneri* serotype 6 is very distantly related to the *S. flexneri* strains described in this study (Table 1) and is therefore excluded from the tree.

The phylogenetic relationships of the *S. flexneri* Type strains showed the somatic antigen structure and phylogenetic relationships were broadly congruent for strains expressing type specific antigens III, IV and V, but not I and II (Figure 1). In addition, serotype 3a was more closely related to the serotype X isolate than isolates expressing serotypes 3b and 3c. Serotype 3c was phylogenetically closely related to serotype 3b but differed phenotypically as it failed to agglutinate with the 3,4 (y) group factor antigen. Serotype 3c is not longer included in the current serotyping scheme [3] as it is very rarely identified (nine isolates submitted to GBRU since 2004).

It has long been reported that the somatic O antigen of *S. flexneri* serotype 6 differs considerably from that of the other *S. flexneri* serotypes and that strains of *S. flexneri* serotype 6 resemble strains of *S. boydii* immunochemically [9]. Consistent with previous studies and phenotypic information, serotype 6 formed an out group from the other *S. flexneri* serotypes sequences (data not shown) [10] being more closely related to *Shigella boydii* CDC 3083–94 (GenBank: CP001063.1); differing by 47 787 SNPs from *S. flexneri* 2a (Table 1) and approximately 7300 SNPs from *S. boydii* CDC 3083–94 (data not shown).

In 1972, colleagues in our laboratory reported a provisional new serotype, designated E1037, frequently submitted to PHE between 2004 and 2013 (276 isolates submitted to GBRU since 2004). Phylogenetically, E1037 is closely related to Serotype 4a (Figure 1). Other groups have supported the extension of the accepted classification scheme to include this novel type [11,12].

The presence of key diagnostic and molecular serotyping genes was also determined. We confirmed the presence of the *ipaH* gene (the target gene for the detection of *Shigella* species in diagnostic PCR assays) in all the PHE Type strains. It was not possible to *de novo* assemble the complete *ipaH* gene in any strain analysed here due to the presence of multiple homologues of *ipaH* in the genome. However, all 16 genomes showed the presence of the entire length of *ipaH* by either BLAST comparison of multiple contigs or mapping to the *S. flexneri* 2a 2457 T reference genome.

The molecular serotyping detailed in Sun *et al*. [3] correlated with the phenotypic data for all isolates tested (Table 1). The provisional type, E1037, was the only Type Strain to contain a copy of the plasmid-mediated seroconverting *Ipt-O* (*opt*) gene [12]. In contrast to the serotype 5 strain described by Sun *et al*. (2012) [3], both PHE serotype 5 Type strains encoded an additional *oac* gene which was intact according to *de novo* assembly and the presence of the *oac* gene was confirmed by PCR [3]. The 5a and 5b serotypes were differentiable by the presence (serotype 5b) or absence (serotype 5a) of *gtrX* (Table 1).

### Future directions

The PHE *S. flexneri* Type strain data set has been used in the validation and evaluation of genotypic and phenotypic assays and has facilitated the study of phylogenetic relationships within this species during outbreak investigations (unpublished observations). Analysis of the genome sequences, in conjunction with the phenotypic serotyping data, provided new insights into this historic strain set. Comparisons with the PCR serotyping scheme highlighted the need to add novel variants [13] in order to maintain a comprehensive collection of relevant Type strains.

## Abbreviations

SNPs: Single nucleotide polymorphisms.

## Competing interests

The authors declare that they have no competing interests.

## Authors’ contributions

AG and CJ performed serology and DNA extraction. DW carried out the DNA sequencing. PA, KB, NRT and TJD analyzed the sequencing data. PA, KB, TJD and CJ contributed to the writing of the manuscript. All authors read and approved the final manuscript.
